# Chemical Synthesis and Characterization of Poly(poly(ethylene glycol) methacrylate)-Grafted CdTe Nanocrystals via RAFT Polymerization for Covalent Immobilization of Adenosine

**DOI:** 10.3390/polym11010077

**Published:** 2019-01-06

**Authors:** Trinh Duy Nguyen, Hieu Vu-Quang, Thanh Sang Vo, Duy Chinh Nguyen, Dai-Viet N. Vo, Dai Hai Nguyen, Kwon Taek Lim, Dai Lam Tran, Long Giang Bach

**Affiliations:** 1 NTT Hi-Tech Institute, Nguyen Tat Thanh University, 300A Nguyen Tat Thanh, District 4, Ho Chi Minh City 755414, Vietnam; ndtrinh@ntt.edu.vn (T.D.N.); vqhieu@ntt.edu.vn (H.V.-Q.); vtsang@ntt.edu.vn (T.S.V.); ndchinh@ntt.edu.vn (D.C.N.); 2 Faculty of Chemical Engineering and Food Technology, Nguyen Tat Thanh University, 300A Nguyen Tat Thanh, District 4, Ho Chi Minh City 755414, Vietnam; 3 Faculty of Chemical & Natural Resources Engineering, University Malaysia Pahang, Lebuhraya Tun Razak, Gambang 26300, Malaysia; vietvo@ump.edu.my; 4 Graduate University of Science and Technology, Vietnam Academy of Science and Technology, 01 Mac Dinh Chi, District 1, Ho Chi Minh 700000, Vietnam; nguyendaihai0511@gmail.com (D.H.N.); trandailam@gmail.com (D.L.T.); 5 Institute of Applied Materials Science, Vietnam Academy of Science and Technology, No.01A TL29 Street, Thanh Loc Ward, District 12, Ho Chi Minh 700000, Vietnam; 6 Department of Display Engineering, Pukyong National University, 599-1 Daeyeon 3-Dong, Nam-Gu, Busan 608-737, Korea; ktlim@pknu.ac.kr; 7 Institute for Tropical Technology, Vietnam Academy of Science and Technology, 18 Hoang Quoc Viet, Cau Giay, Hanoi 10072, Vietnam

**Keywords:** CdTe quantum dots, poly(poly(ethylene glycol methacrylate), SI-RAFT, adenosine, covalent immobilization

## Abstract

This paper describes the functionalization of poly(poly(ethylene glycol) methacrylate) (PPEGMA)-grafted CdTe (PPEGMA-*g*-CdTe) quantum dots (QDs) via surface-initiated reversible addition–fragmentation chain transfer (SI-RAFT) polymerization for immobilization of adenosine. Initially, the hydroxyl-coated CdTe QDs, synthesized using 2-mercaptoethanol (ME) as a capping agent, were coupled with a RAFT agent, *S*-benzyl *S*′-trimethoxysilylpropyltrithiocarbonate (BTPT), through a condensation reaction. Then, 2,2′-azobisisobutyronitrile (AIBN) was used to successfully initiate in situ RAFT polymerization to generate PPEGMA-*g*-CdTe nanocomposites. Adenosine-above-PPEGMA-grafted CdTe (Ado-*i*-PPEGMA-*g*-CdTe) hybrids were formed by the polymer shell, which had successfully undergone bioconjugation and postfunctionalization by adenosine (as a nucleoside). Fourier transform infrared (FT-IR) spectrophotometry, energy-dispersive X-ray (EDX) spectroscopy, thermogravimetric analysis (TGA), X-ray photoelectron spectroscopy (XPS), and transmission electron microscopy results indicated that a robust covalent bond was created between the organic PPEGMA part, cadmium telluride (CdTe) QDs, and the adenosine conjugate. The optical properties of the PPEGMA-*g*-CdTe and Ado-*i*-PPEGMA-*g*-CdTe hybrids were investigated by photoluminescence (PL) spectroscopy, and the results suggest that they have a great potential for application as optimal materials in biomedicine.

## 1. Introduction

Semiconductor nanocrystals, such as quantum dots (QDs), have recently attracted much attention in biological fields because of their advantageous properties, including high photostability, sharp emission, wide absorption, and especially high photoluminescence (PL) quantum yield [1,2,3,4,5,6,7,8]. Hence, QD nanocrystals are broadly employed as a fluorescence tool for biological labeling, targeted drug delivery, photodynamic treatment, nanodiagnostics, and imaging. In recent years, biological ligand conjugates of low-cytotoxic agents, such as folic acid, biopolymers, peptides, and sugars, have been used to prepare QD nanoplatforms to incorporate many functions [9,10,11,12,13,14,15]. Embedding QDs in a steady polymer coating inhibits the corrosion and cytotoxicity of the prepared capsules. Surface modifications on QDs have also been employed in the design of new probes with integrated functionalities to study protein trafficking and target cell surface receptors [16,17,18,19,20,21].

Because of the wide range of applications, the expansion of metal and semiconductor–polymer nanocomposites has created new opportunities and challenges for future technologies. A polymer is an excellent choice for a matrix material because it enables simple processing of the poly(ethylene glycol) (PEG)—based nanocomposites into technologically effective forms. In particular, PEG possesses many important properties that can be used in the pharmaceutical field, such as significant inertness to cell and protein adhesion, good biocompatibility, low toxicity, nonimmunogenicity, and high water solubility [22,23,24,25,26,27,28]. Moreover, by introducing PEG functionality to the surface, the biocompatibility of nanoparticles can be improved. Poly(ethylene glycol) brushes not only provide a nonadhesive characteristic, but their terminal hydroxyl groups can also be employed for biomolecule immobilization, which has many useful applications in medicine and biotechnology. Microarraying and biosensing devices have proven that there is much advantage in the development of biological agents; in addition, biological interactions, such as hybrid DNA and similar protein–protein and protein–antibody interaction interfaces, have also been recognized. Adenosine is an endogenous purine nucleoside that is created in all living systems, and it plays an important role as a structural component of important biological molecules, such as RNA and DNA. As it is the major molecule of adenosine diphosphate, adenosine monophosphate, and adenosine triphosphate, it transports chemical energy within cells for metabolism and expresses the essential transmission functions in the central nervous system [29,30,31,32,33,34,35].

To adjust the surface properties of the solid substrate, the tethering of the polymer brush on the substrate is considered as an efficient approach. Reversible addition–fragmentation chain transfer (RAFT) polymerization is one of the techniques used to prepare polymer brushes, and it is seen as a “controlled” radical polymerization route. It involves a single route in the preparation of dense brushes of low polydispersity, well-defined components and thickness, and controlled architecture [36,37,38,39,40,41,42]. The RAFT polymerization, when compared with other living polymerization techniques, is compatible with various functions (containing reactive functional groups) in monomers, making it an effective synthetic tool for potential tailored designs and in preparing polymers with fluorescent labels, polymer–drug conjugates, and novel polymeric bioconjugates. Many biocompatible polymers, such as poly(2-hydroxyethyl methacrylate) (PHEMA), poly(poly(ethylene glycol) methyl ether monomethacrylate) (PPEGMA), and poly(allyl methacrylate) (polyAMA), have been employed to decorate nanomaterials via RAFT polymerization in order to prepare new materials for different purposes [43,44,45,46,47,48,49].

Taking the above consideration into account, we have developed a new strategy to prepare multifunctional, stable QDs. In this study, cadmium telluride (CdTe) QDs were coated with poly(poly(ethylene glycol) methacrylate) (PPEGMA) brushes via surface-initiated RAFT (SI-RAFT) polymerization to be used in fluorescent probes for biomedical applications. Surface biocompatibility was ensured by attaching adenosine to one end of the PEG branches using the *N*,*N*′-disuccinimidyl carbonate (DSC) as a biofunctional linker.

## 2. Experimental Section

### 2.1. Materials

Poly(ethylene glycol) methacrylate (PEGMA; *M*_n_ = 360) was used after removing the inhibitor by basic alumina column chromatography. Prior to use, 2,2′-azobisisobutyronitrile (AIBN) was refined from methanol by recrystallization. Sodium tellurite (Na_2_TeO_3_), sodium methoxide, 3-(mercaptopropyl)trimethoxysilane, 2-mercaptoethanol (ME), CdCl_2_·2.5H_2_O, C_S2_, benzyl bromide, DSC, adenosine, *N*,*N*′-Dimethylformamide (DMF), 4-(dimethylamino)pyridine, anhydrous methanol, and dichloromethane were purchased from Sigma-Aldrich (St. Louis, MO, USA) and used as received. All chemicals used were of analytical grade, and distilled water was used during the experiments.

### 2.2. Preparation of S-Benzyl S′-Trimethoxysilylpropyltrithiocarbonate

The preparation of S-benzyl S′-trimethoxysilylpropyltrithiocarbonate (BTPT) was as follows. First, 0.81 g of sodium methoxide was dissolved in anhydrous methanol (5 mL), and the solution was then added dropwise to a mixture containing 3.10 g (3-mercaptopropyl)trimethoxysilane in anhydrous methanol (25 mL) under nitrogen. The solution was stirred for 1 h for the reaction to occur. Subsequently, 1.5 g of CS_2_ was added into the above solution while stirring at room temperature. After 5 h, 2.62 g of benzyl bromide was added and stirred overnight under nitrogen. When the mixture became concentrated, it was diluted with dichloromethane, which was then filtered off. The mixture was further concentrated with reduced pressure until the mass was constant. Orange oil (BTPT, 3.6 g, 10 mmol) was acquired and purified. BTPT: ^1^H NMR (CDCl_3_): 3.54 (s, CH_3_O), 7.34 (m, PhH), 4.61 (s, CH_2_PhH), 1.77 (m, CH_2_), 3.40 (t, CH_2_S), 0.77 (t, CH_2_Si).

### 2.3. Synthesis of Hydroxyl-Coated CdTe Nanocrystals

First, CdCl_2_·2.5H_2_O was dissolved in 50 mL of distilled water, and an appropriate amount of ME was added while stirring. Then, the pH was adjusted to 9 by adding 1 M NaOH solution. To obtain a yellowish-brown solution of ME-covered CdTe nanocrystals, a suitable amount of Na_2_TeO_3_ was dissolved in distilled water (50 mL), and the mixture was then vigorously stirred with CdCl_2_·2.5H_2_O solution at 90 °C for 5 h.

### 2.4. Immobilization of Chain Transfer Agent on CdTe QD Surface

The CdTe-OH QDs (1.0 g) were silanized by BTPT (1 g) upon stirring them in DMF. The mixture was then stirred vigorously with nitrogen for 12 h at 110 °C and washed with toluene to remove the free BTPT. The BTPT-tethered CdTe nanocrystals were rinsed five times with fresh DMF and dried under vacuum at 30 °C for 2 days.

### 2.5. Grafting of Poly(Ethylene glycol) Methacrylate Brushes on CdTe QDs by SI-RAFT

The PPEGMA-grafted CdTe (PPEGMA-*g*-CdTe) nanocomposites were synthesized via one-step RAFT polymerization with RAFT agent-immobilized magnetic nanoparticles (0.10 g), PEGMA (1.00 g), and AIBN (0.01 g) in 2 mL DMF inside a three-neck glass reactor attached to a stirrer, thermocouples, and a reflux condenser and then stirred in nitrogen at 70 °C. To obtain PPEGMA-*g*-CdTe hybrids, the mixture was stirred vigorously in nitrogen for further 4 h at 70 °C. Then, PPEGMA-coated CdTe was separated from the suspension and rinsed with methanol several times. Finally, the material was dried at 40 °C under a vacuum oven to obtain the PPEGMA-*g*-CdTe nanocomposites.

### 2.6. Conjugation of Adenosine onto PPEGMA-*g*-CdTe Nanocomposites

The terminal hydroxyl species of PPEGMA were initiated with DSC to conjugate the PPEGMA-*g*-CdTe QDs with adenosine. Next, 0.5 mL of DMF solution, prepared by DSC (0.1 M) and 4-(dimethylamino)pyridine (0.1 M), was placed in a vial and used to dissolve 1.2 mg of the PPEGMA-*g*-CdTe nanocomposites. The reaction was activated at an ambient temperature for 3 h. To obtain DSC-*f*-PPEGMA-*g*-CdTe QDs, the nanoparticles were first washed with DMF to remove the adsorbed reagents and dried at 40 °C in a vacuum oven for a day. Afterward, 5.0 g of adenosine was dissolved in 5 mL of phosphate-buffered saline (PBS) solution, which was then sonicated to acquire a uniform solution. Subsequently, 0.1 g of DSC-*f*-PPEGMA-*g*-CdTe was added to 10 mL of PBS solution to give a suspension, which was then added to the adenosine solution at ambient temperature. After continuously stirring for 4 h, the samples were collected and washed several times with PBS, then dried at 40 °C under a vacuum oven to acquire the adenosine-above-PPEGMA-grafted CdTe (Ado-*i*-PPEGMA-*g*-CdTe) nanohybrids.

### 2.7. Measurements

Under a frequency range of 4000–400 cm^−1^, the changes in the surface chemical bonding of the functionalized QDs were captured by Fourier transform infrared (FT-IR) spectrophotometry using a BOMEM Hartmann & Braun FT-IR spectrometer (Frankfurt, Germany). The surface component was inspected using X-ray photoelectron spectroscopy (XPS) (Thermo VG Multilab 2000, Waltham, MA, USA) with Al Kα radiation in an ultrahigh vacuum. The elemental examination of the hybrids was realized using a field emission scanning electron microscope furnished with an energy-dispersive X-ray (EDX) spectrometer (Hitachi JEOL-JSM-6700F system, Tokyo, Japan). At a constant stimulation wavelength of 360 nm, PL spectra were observed by an F-4500 spectrofluorometer (Hitachi).

## 3. Results and Discussion

**Scheme 1:**
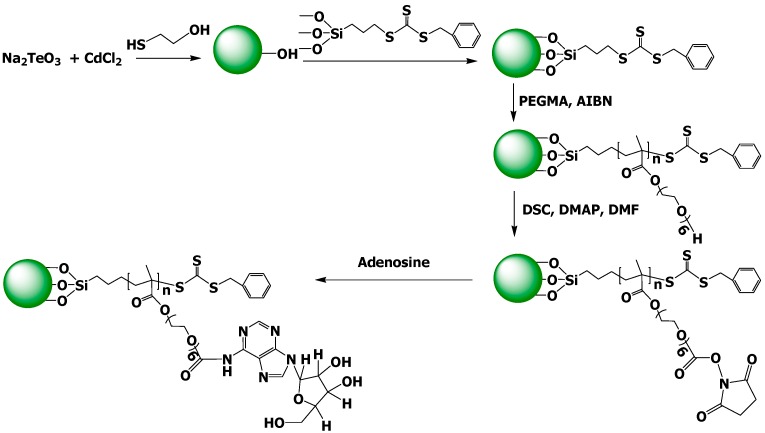
Route for the synthesis of poly(poly(ethylene glycol) methacrylate) (PPEGMA)-coated CdTe quantum dots (QDs) via reversible addition–fragmentation chain transfer (RAFT) polymerization and subsequent conjugation of adenosine.

In this research, Ado-*i*-PPEGMA-*g*-CdTe QDs was obtained by selectively initiating the last hydroxyl (–OH) species derived from PEG molecules with DSC, followed by a conjugation reaction to generate N–H bonds (Scheme 1). First, ME containing the thiol group was used as the organic ligand, and hydroxyl-covered CdTe was prepared in the presence of ME by an in-place reaction of cadmium and tellurium ions. Biocompatible polymer-grafted nanohybrid PPEGMA-*g*-CdTe was prepared by the surface-initiated RAFT of PEGMA from the surface of strategic RAFT agent CdTe-BTPT QDs. Then, the coupling of adenosine at the chain end of PEG branches of PPEGMA-*g*-CdTe nanocomposites via the *N*,*N*′-disuccinimidyl carbonate as a biofunctional linker resulted in Ado-*i*-PPEGMA-*g*-CdTe multifunctional nanohybrids, which could be applicable in biomedicine as new fluorescent probes. The nanohybrids were investigated by respective analytical techniques. For analyses, FT-IR spectroscopy, XPS, and EDX methods were used to determine the properties of RAFT agents-immobilized CdTe QDs.

**Figure 1:**
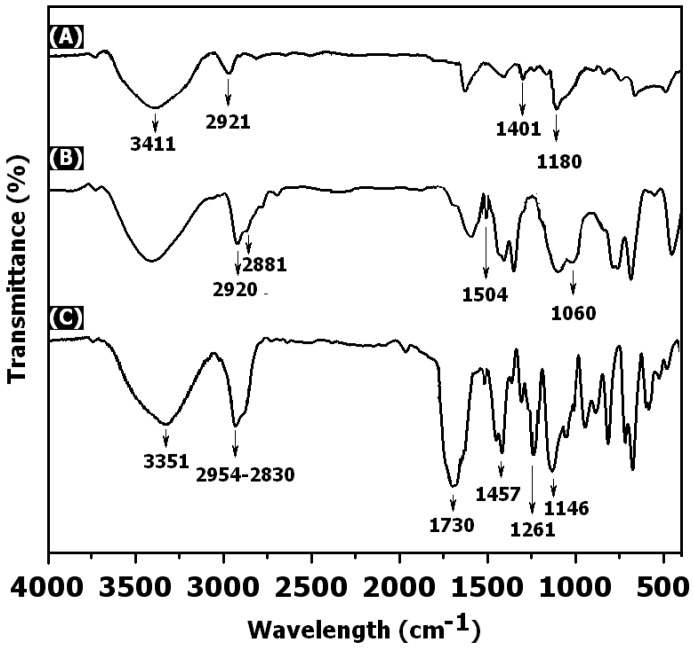
Fourier transform infrared (FT-IR) spectra of (**A**) CdTe-OH, (**B**) CdTe- S-benzyl S′-trimethoxysilylpropyltrithiocarbonate (BTPT), and (**C**) PPEGMA-grafted CdTe (PPEGMA-*g*-CdTe).

Figure 1 displays the FT-IR spectrum of ME-capped CdTe QDs and RAFT agent-functionalized CdTe QDs. The characteristic absorption wavenumber of around 3411 cm^−1^ was due to the –OH vibrations. Meanwhile, several broad peaks at 2921, 1405, and 1180 cm^−1^ were derived by stretching the vibrations of the sp3 methyl species and ethers of ME attached to the CdTe QD surface (Figure 1A). The band at 2538 cm^−1^ responsible for –SH was not observed because of the covalent bonds between thiols and Cd on the CdTe QD surface. Compared with CdTe-OH, the FT-IR spectrum of CdTe-BTPT (Figure 1B) clearly showed the fragments of propyl groups, found at 2920 and 2881 cm^−1^, while a footprint shape at 1504 cm^−1^ was accountable for aromatic cycles. Furthermore, Si–O–Si bonds had an adsorption peak at 1060 cm^−1^ because of the presence of a polysiloxane film on the CdTe surface as the silane groups experienced a self-condensing reaction.

**Figure 2:**
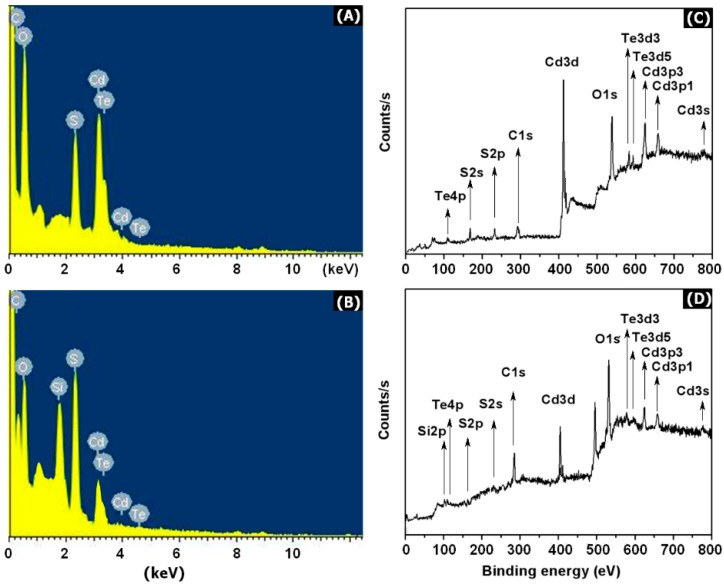
Energy-dispersive X-ray (EDX) spectra of (**A**) CdTe-OH and (**B**) CdTe-BTPT. X-ray photoelectron spectroscopy (XPS) spectra of wide-scan of (**C**) CdTe-OH, and (**D**) CdTe-BTPT.

The EDX spectrum of CdTe-OH showed the presence of Cd, Te, S, C, and O in CdTe-OH (Figure 2A). The EDX spectrum of CdTe-BTPT revealed that a small signal was assigned to silicon atoms on their exterior (Figure 2B). Considering the above findings, the RAFT agent was more likely to covalent bond onto the CdTe QD surfaces. Additionally, the typical chemical bonds of CdTe-OH and CdTe-BTPT surfaces were measured by XPS analysis (Figure 2C,D). Figure 2D shows the attachment of the RAFT agent to the CdTe exterior represented by the Cd3s, Cd3p1, Cd3p3, Te3d3, Te3d5, O1s, Cd3d, C1, S2, S2p, Te4p, and Si2p signals with intensities of 722.9, 658.6, 624.5, 593.7, 583.1, 538.6, 412.21, 292.2, 232.5, 168.6, 116.1, and 102.4, respectively. The occurrence of the Si signal in the broad spectrum of the RAFT agent was due to the attachment of a RAFT-silane coupling agent (BTPT) on CdTe QDs monolayer via condensation reactions.

**Figure 3:**
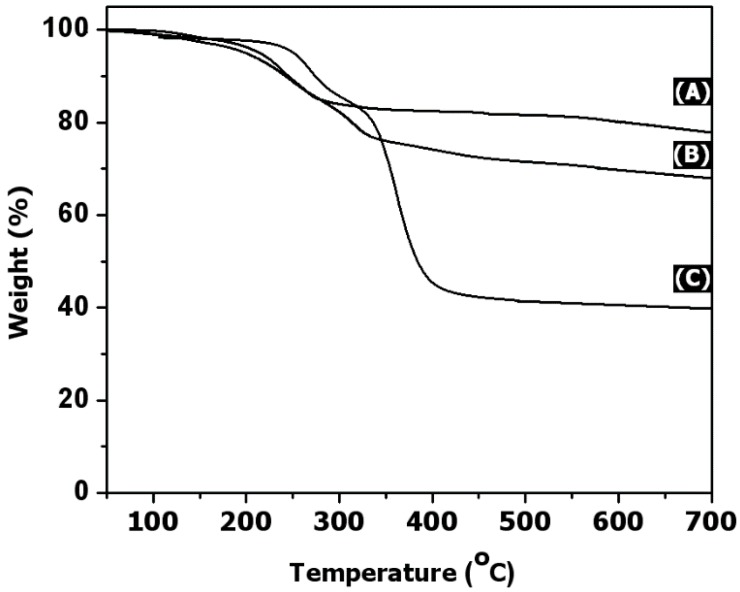
Thermogravimetric analysis (TGA) scans of (**A**) CdTe-OH, (**B**) CdTe-BTPT, and (**C**) PPEGMA-*g*-CdTe.

Thermogravimetric analysis (TGA) curves showed the amount of RAFT agents immobilized to CdTe QDs as well as the initial and final degradation temperatures. From 100–7000 °C, the CdTe-OH sample experienced a weight loss of around 20% of its initial weight because of dehydration on the surface and destruction of hydroxyl groups in the CdTe-OH structure. From the TGA results (Figure 3B), the CdTe-BTPT was approximated to be 31.7%, resulting in the amount of the grafted RAFT agents of approximately 10.9%.

The surface grafting of RAFT is expected to promote the anchoring of PPEGMA chains onto the CdTe surface. As can be seen in Figure 1C, the results of FT-IR analysis displayed peaks of 3351 (–O–H), 2830–2954 (aromatic C–H), 1730 (C=O groups), 1457 (aliphatic C–H groups), 1263 (bending and rocking C–H groups), and 1146 cm^−1^ (ether species). This showed that PPEGMA was covalently attached to the exterior of the CdTe QDs.

Moreover, TGA was utilized to expose the CdTe QD ingredients on the PPEGMA layer. Between 285 and 430 °C, the TGA curve of PPEGMA-*g*-CdTe nanocomposites displayed a massive weight loss because of the presence of the peripheral and significant polymer sequences. Figure 3C shows the TGA scan of the surface-grafted polymers. Because of the existence of CdTe QDs, the polymers produced a char yield up to 32.7% at 700 °C. The results showed that the thermal constancy of PPEGMA-*g*-CdTe was improved through the formation of well-dispersed nanohybrids.

**Figure 4:**
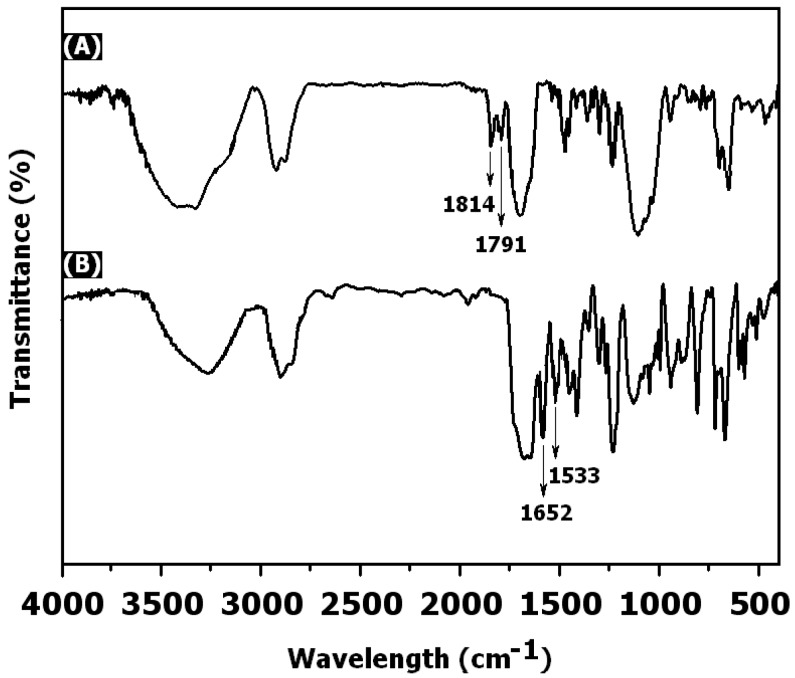
FT-IR spectra of (**A**) DSC-f-PPEGMA-*g*-CdTe, (**B**) Adenosine-above-PPEGMA-grafted CdTe (Ado-*i*-PPEGMA-*g*-CdTe) hybrids.

The combination of O–H groups to the edge chains of a PEG-based polymer has become a new application for biomolecule conjugates (eg., antibacterial peptides and enzymes). In this study, we used DSC to activate the hydroxyl groups of the PPEGMA brushes to create DSC-*f*-PPEGMA-*g*-CdTe QD surface. The FT-IR spectrum analysis (Figure 4A) showed peaks at 1814 and 1791 cm^−1^, which describe the C=O stretching vibration of the NHS group, confirming that the hydroxyl groups had been activated. After activation, we observed the combination of the activated PPEGMA-recovered CdTe with bioactive molecules. The activated PPEGMA shells formed the amide bond with a main amino group of the adenosine substance. A change in FT-IR spectrum was observed after the functionalization process of DSC-*f*-PPEGMA-*g*-CdTe QDs with adenosine compounds. As shown in Figure 4, the peaks at 1652 and 1533 cm^−1^ could be attributed to the amide in adenosine compounds, while the two peaks at around 1814 and 1791 cm^−1^ showed that the NHS species were absent, confirming the bonds in adenosine. This verified that adenosine compounds were grafted on the outer of CdTe via covalent modification.

**Figure 5:**
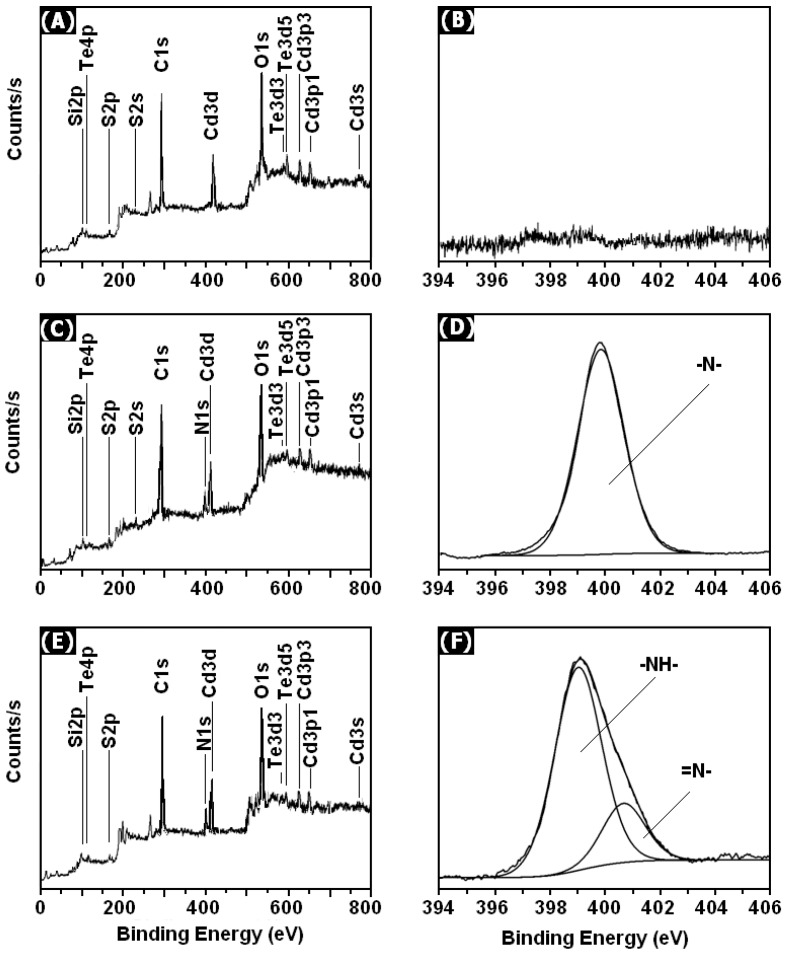
Wide scans and N1s core-level spectra of (**A**,**B**) PPEGMA-*g*-CdTe, (**C**,**D**) DSC-*f*-PPEGMA-*g*-CdTe, and (**E**,**F**) Ado-*i*-PPEGMA-*g*-CdTe nanohybrids, respectively.

The surface chemical ingredient of the Ado-*i*-PPEGMA-*g*-CdTe hybrids was analyzed using XPS observations. The corresponding vast-scan spectrum and N1s core-level spectra of the DSC-*f*-PPEGMA-*g*-CdTe surface are shown in Figure 5. The effective combination with DSC was demonstrated by the presence of the N1s signal background in the broad scanning spectrum of the DSC-*f*-PPEGMA-*g*-CdTe exterior (Figure 5B) compared to the CdTe-*g*-PEGEGMA exterior. The N1s core level spectrum with a binding energy of 399.8 eV was ascribed to the amine groups in the DSC. The N1s spectral line shapes of Ado-*i*-PPEGMA-*g*-CdTe surface were dissimilar to those of DSC-*f*-PPEGMA-*g*-CdTe. Figure 5F indicates that the N1s spectrum included amine (339.6 eV) and imine (397.5 eV), combined with adenosine. Thus, the combination of the Ado-*i*-PPEGMA-*g*-CdTe exterior with the crowded PPEGMA polymer brushes and adenosine was successfully admixed.

**Figure 6:**
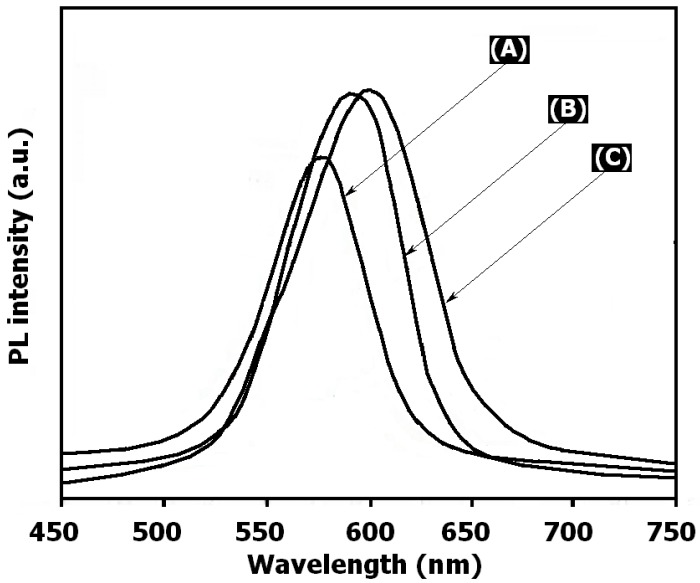
Photoluminescence (PL) spectra (excited at 380 nm) of (**A**) CdTe-OH QDs, (**B**) PPEGMA-*g*-CdTe, and (**C**) Ado-*i*-PPEGMA-*g*-CdTe nanohybrids.

The specific binding of adenosine-conjugated PPEGMA-coated CdTe QDs was characterized by fluorescence spectroscopy. For clear observation, the PL emission spectra of the CdTe-OH, PPEGMA-*g*-CdTe, and Ado-*i*-PPEGMA-*g*-CdTe hybrids were compared. At 572 nm, the CdTe-OH spectrum showed a potent emission peak (Figure 6A), which exhibited a sharp excitonic emission feature. After clouding with PPEGMA, the wavelength of the PPEGMA-*g*-CdTe culmination emission peak (590 nm) was longer than that of the CdTe-OH peak (Figure 6B). Upon covalent immobilization of PPEGMA onto the CdTe surface, the PL intensity of the nanocomposites increased in comparison with ME-capped CdTe QDs. It is speculated that the covalently grafted PPEGMA acted as an active barrier that protected the CdTe QD surface and prevented the photogeneration of carriers outside the captured CdTe QDs. As a result, the emission intensity was enhanced. After PPEGMA-*g*-CdTe bonded with adenosine, the feature absorption of Ado-*i*-PPEGMA-*g*-CdTe hybrids led to a higher emission at 600 nm wavelength (Figure 6). The Ado-*i*-PPEGMA-*g*-CdTe nanohybrids showed a strong, broad emission around 600 nm, which might be due to the strong covalent immobilization of adenosine to the surface of PPEGMA-*g*-CdTe. It is widely believed that the large peak in the observable domain is related to structural blemishes, such as exterior pits on the QDs, selenium vacancies, and surface traps on the QDs. In the case of CdTe QDs, surface states such as dangling bonds are usually involved in nonradiative processes, while Te^2−^ ions provide a critical pathway for the visible emission band. Because the PPEGMA and adenosine immobilization reduce the density of surface dangling bonds and Te^2−^ ions, the possibility of nonradiative transitions could be further reduced, thereby increasing the probability of visible emission.

## 4. Conclusions

In this study, an environmentally friendly bioconjugation of PPEGMA-covered CdTe QDs by SI-RAFT polymerization for biomolecule conjugates was investigated. Hydroxyl-containing CdTe QDs were initially synthesized using ME and then anchored to *S*-benzyl *S*′-trimethoxysilylpropyltrithiocarbonate through a condensation reaction. Poly(poly(ethylene glycol methacrylate)-grafted CdTe QDs consisting of covalent bonds were successfully fabricated via SI-RAFT polymerization. Results of FT-IR, XPS, and EDX analyses showed that a strong covalent link was created between polymer moieties, RAFT-anchored, and ME-capped CdTe QDs. Thermogravimeteric analysis showed that the thermal stability of the hybrids had improved in comparison to CdTe, and the grafting density of PPEGMA onto CdTe was reasonable. The –OH groups of the PPEGMA shell-grafting CdTe QDs were triggered by DSC, followed by the tie-up of adenosine at the chain ends of PEG branches. The surface functionalization of CdTe QDs by PPEGMA and adenosine displayed a red shift in the photoluminescence.
